# Stylish UV-C lamp for disinfecting household objects

**DOI:** 10.1093/eurpub/ckac131.020

**Published:** 2022-10-25

**Authors:** R Iannaccone, N Nante, I De Palma, D Amodeo, G Messina

**Affiliations:** 1 Post Graduate School of Public Health, University of Siena, Siena, Italy; 2 Department of Molecular and Developmental Medicine, University of Siena, Siena, Italy; 3 Department of Medical Biotechnologies, University of Siena, Siena, Italy

## Abstract

**Background:**

Considering the current pandemic situation, the growing problem of antibiotic resistance and the increase in healthcare costs, attention to daily disinfection is becoming increasingly important. This study aimed to evaluate the bactericidal efficacy of a modern and stylish UV-C device designed for the home environment.

**Methods:**

The experimental study was conducted between July-August 2020 on four bacterial strains: Staphylococcus aureus, Salmonella typhymurium, Klebsiella pneumoniae and Escherichia coli. The UV-C device consist of a protective dome with a reflective coating, a UV-C lamp (placed in the device base) and three reflective holders. Different positions and exposure times were tested using two different carriers holder for the bacterial inoculum (plastic and stainless steel) to estimate the germicidal efficency related to UV-C lamp exposure, with direct and reflected (from the dome coating) light.

**Results:**

The experiment showed that the higher bacterial inactivation effect (3.5 to 7 log10) was achieved for all four strains at 3 minutes, but even at 1 minute, there is a marked reduction in the bacterial load with the only exception of Klebsiella pneumoniae. After 45 and 30 seconds, steel carriers contaminated by Escherichia coli and Staphylococcus aureus on the opposite side of the UV-C source showed significant reductions in the range between 99 and 99,9%.

**Conclusions:**

The device has proven to be effective for the disinfection of various everyday objects placed into the lamp and introduces beauty to the household environment.

**Key messages:**

• In this study, UV-C device proved to be a valuable tool for disinfecting household items and enhancing safety for everyday health.

• UV-C device proved to be a valuable tool for disinfecting household items and enhancing safety for everyday health.

